# Mining the bladder cancer-associated genes by an integrated strategy for the construction and analysis of differential co-expression networks

**DOI:** 10.1186/1471-2164-16-S3-S4

**Published:** 2015-01-29

**Authors:** Su-Ping Deng, Lin Zhu, De-Shuang Huang

**Affiliations:** 1 College of Electronics and Information Engineering, Tongji University, Caoan Road, Shanghai, China

## Abstract

**Background:**

Bladder cancer is the most common malignant tumor of the urinary system and it is a heterogeneous disease with both superficial and invasive growth. However, its aetiological agent is still unclear. And it is indispensable to find key genes or modules causing the bladder cancer. Based on gene expression microarray datasets, constructing differential co-expression networks (DCNs) is an important method to investigate diseases and there have been some relevant good tools such as R package 'WGCNA', 'DCGL'.

**Results:**

Employing an integrated strategy, 36 up-regulated differentially expressed genes (DEGs) and 356 down-regulated DEGs were selected and main functions of those DEGs are cellular physiological precess(24 up-regulated DEGs; 167 down-regulated DEGs) and cellular metabolism (19 up-regulated DEGs; 104 down-regulated DEGs). The up-regulated DEGs are mainly involved in the the pathways related to "metabolism". By comparing two DCNs between the normal and cancer states, we found some great changes in hub genes and topological structure, which suggest that the modules of two different DCNs change a lot. Especially, we screened some hub genes of a differential subnetwork between the normal and the cancer states and then do bioinformatics analysis for them.

**Conclusions:**

Through constructing and analyzing two differential co-expression networks at different states using the screened DEGs, we found some hub genes associated with the bladder cancer. The results of the bioinformatics analysis for those hub genes will support the biological experiments and the further treatment of the bladder cancer.

## Background

The morbidity of bladder cancer is in the first place among the cancers of urinary system. The bladder cancer cells can spread by breaking away from the original tumor. They can spread through the blood vessels to the liver, lungs and bones. However, its causes are not yet clear. The bladder cancer is a heterogeneous disease that shows both superficial and invasive growth [1,2]. Superficial tumors frequently recur and may progress to invasive growth. A part that warrants better treatment regimes bladder cancer is also a good model system to study tumor initiation and progression. To gain insights into the molecular biology of these processes, we performed gene expression analyses to get some important information about bladder cancer-associated genes.

Systems biology is an emerging approach applied to biomedical and biological scientific research. It is a biology-based inter-disciplinary field of study that focuses on complex interactions within biological systems, using a holistic approach to biological and biomedical research [3-5]. Network biology is a new way of representation and analysis of biological information processing, which understands life as a network. In fact, the network biology is a branch of the systems biology.

Differential co-expression network (DCN) is one of biological networks. A gene co-expression network has emerged as a novel holistic approach for microarray analysis [6-9]. Stuart et al. [6] and Bergmannet al.[7] separately constructed the gene co-expression network that connected genes whose expression profiles were similar across different organisms. A human network was analyzed by Leeet al. [8] with functional grouping and cluster analysis. van Noort et al. [9] demonstrated the small-world and scale-free architecture of the yeast co-expression network. They showed that functionally related genes are frequently co-expressed across organisms constituting conserved transcription modules.

We wanted to explore transcriptional changes in terms of gene interactions rather than at the level of individual genes. In the end, we constructed two gene co-expression networks and sought to find cancer-induced changes in the network. The identification of co-expressed pairs in tumor and normal tissues led to the construction of two distinct networks that represent tumor and normal states, respectively. We expected that biological changes would be reflected in transcriptional changes, which could be identified by comparing the two co-expression networks. In the transcriptome analysis, differential co-expression analysis (DCEA) is emerging as a unique complement to traditional differential expression analysis. DCEA investigates differences in gene interconnection by calculating the expression correlation changes of gene pairs between two conditions. The rationale behind differential co-expression analysis is that changes in gene co-expression patterns between two contrasting phenotypes (e.g., healthy and disease) provide hints regarding the disrupted regulatory relationships or affected regulatory subnetworks specific to the phenotype of interest (in this case, the disease phenotype). Therefore, among the many growing directions of DCEA, there is the so-called "differential regulation analysis"(DRA), which integrates the transcription factor (TF)-to-target information to probe upstream regulatory events that account for the observed co-expression changes. Recently, many researchers have integrated differential co-expression and differential expression concepts to propose a novel Regulatory Impact Factor (RIF) that can be used to prioritize disease-causative TFs [10,11].

In addition, a lot of researchers have begun to perform differential co-expression analyses of microRNAs [12,13]. Currently, some tools have been developed for differential expression analysis based on microarray, such as R packages "LIMMA"[14], "SAMR"[15], "WGCNA"[16] and so on.

In our study, we collected microarray datasets of bladder cancer from GEO http://www.ncbi.nlm.nih.gov/geo/ to analyze the datasets by an integrated strategy including some functions of SAMR[15], WGCNA[16], Cytoscape[17] and other packages. We selected some DEGs at two different state (normal and cancer) and constructed two DCNs. Through the comparisons between two DCNs of two different states, we found some hub genes associated with the bladder cancer.

## Results

The datasets of gene expression Affymetrix microarray of bladder cancer [GEO: GSE3167] were download from the GEO database of NCBI[18]. It has 18 samples, [GSM71019-GSM71027] are from the normal tissues and the other 9 samples are from the cancer tissues. The datasets were processed by an integrated strategy. Some DEGs were selected and constructed two DCNs. In the end, simple analysis was applied to the two DCNs and some hub genes were found through comparing two DCNs at different states.

### Normalization of the microarray datasets

**Figure 1:**
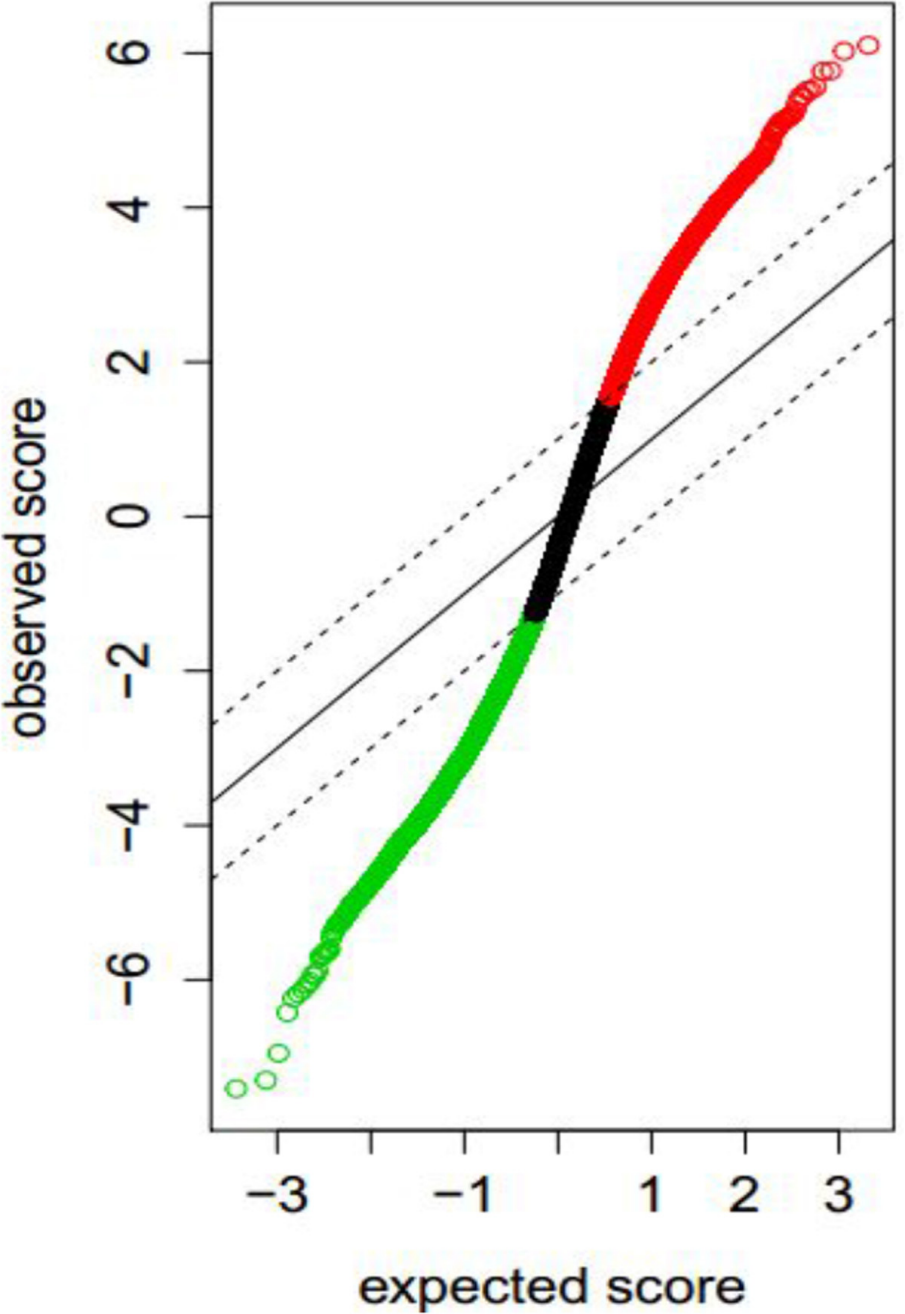
**The Q-Q plot of the expression datasets**.

In order to get high-quality and strong-expression genes for the convenience of the following data processing, we normalized the microarray dataset using medians(Additional file 1 shows the comparison between before and after normalization). After normalization, the expression values are in the better order. We also discovered the distribution of the expression datasets (Figure 1).

### Selection and bioinformatical analysis of DEGs

After preprocessing the microarray datasets including the above normalization, some DEGs were selected using the R package "SAMR" (nperms(Number of permutations used to estimate false discovery rates) = 100; del (Value of delta to define cutoff rule) = 2.5). 36 up-regulated genes and 356 down-regulated genes were picked out (See Additional file 2).

**Figure 2:**
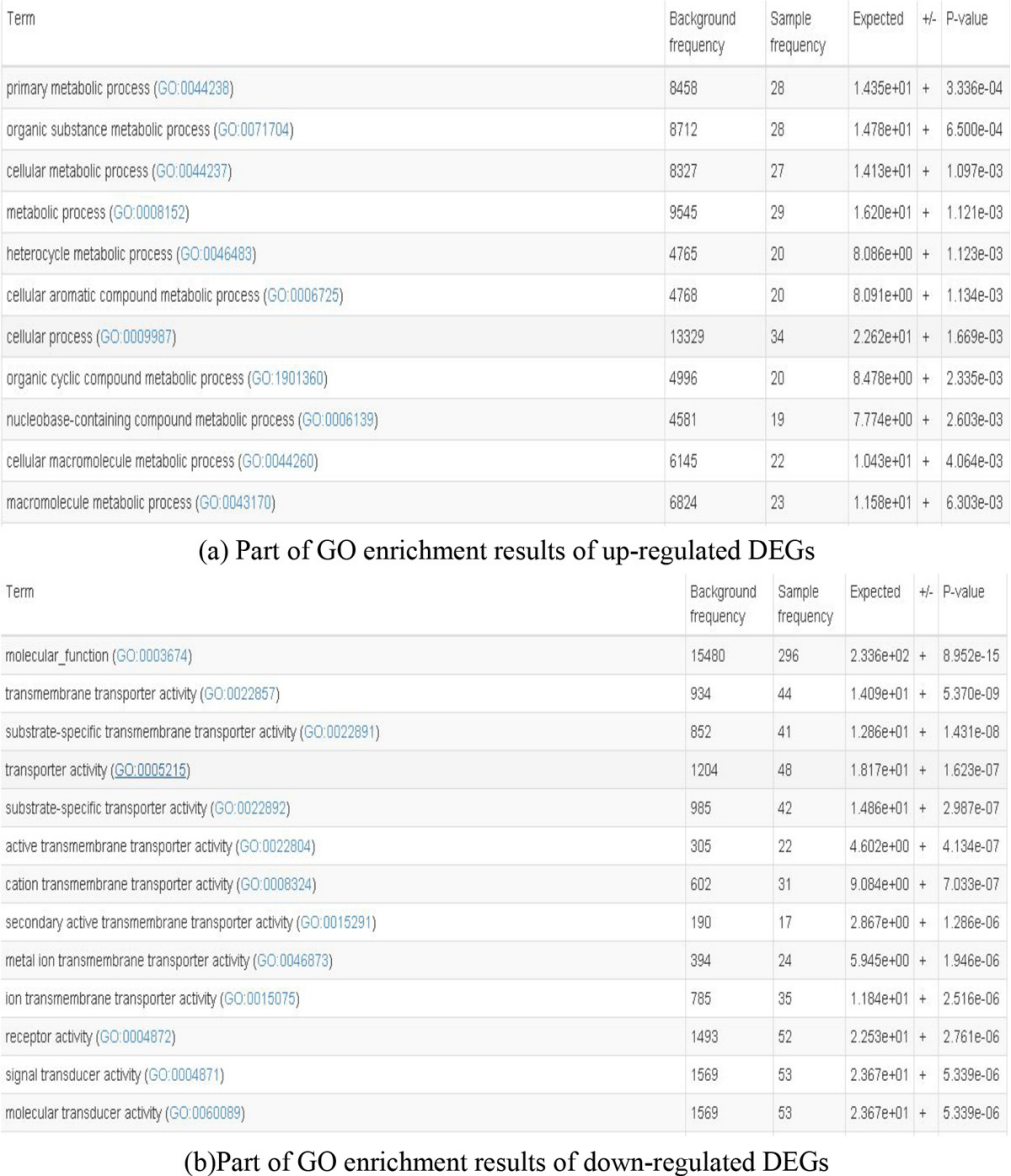
**Part of GO function enrichment results of DEGs associated the bladder cancer**.

Next, we did bioinformatics analysis of those DEGs including GO function enrichment using a online tool AmiGO[19]. A part of GO enrichment results are showed in Figure 2. From the GO function enrichment results, we can easily find that the main functions of the up-regulated DEGs are nitrogen compound metabolic process (GO:0006807)(20 genes), heterocycle metabolic process (GO:0046483) (20 genes), cellular aromatic compound metabolic process (GO:0006725) (20 genes) and regulation of metabolic process (GO:0019222). And the down-regulated DEGs mostly involve ion binding (GO:0043167) (158 genes),multicellular organismal process(GO:0032501)(127 genes), single-multicellular organisms process(GO:0044707) (121 genes) and response to stimulus (GO:0050896) (158 genes) etc.

**Figure 3:**
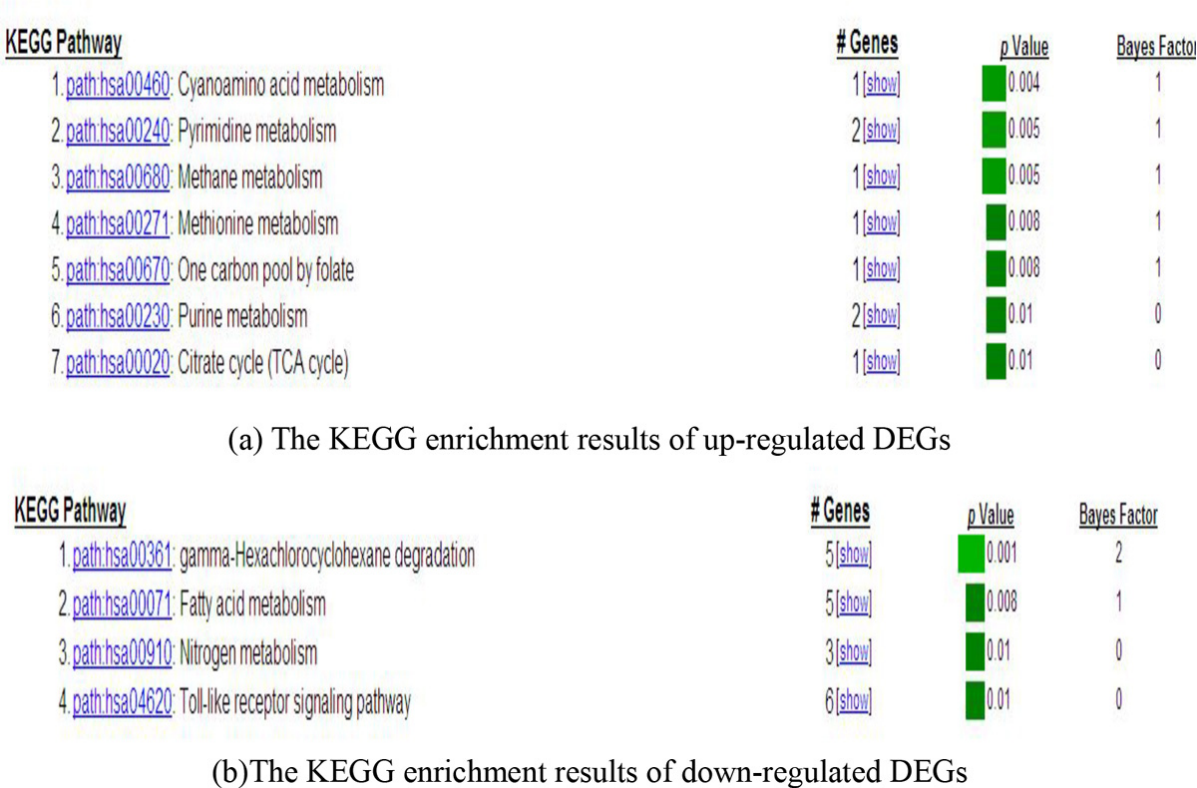
**The KEGG enrichment results of DEGs associated with the bladder**.

We used a online tool GATHER[20] to do pathway enrichment (Figure 3). The up-regulated DEGs mostly involve the pathways related to "metabolism". However, the down-regulated DEGs are included in toll-like receptor signaling pathway, gamma-hexachlorocyclohexane degradation besides two metabolism-associated pathways.

**Figure 4:**
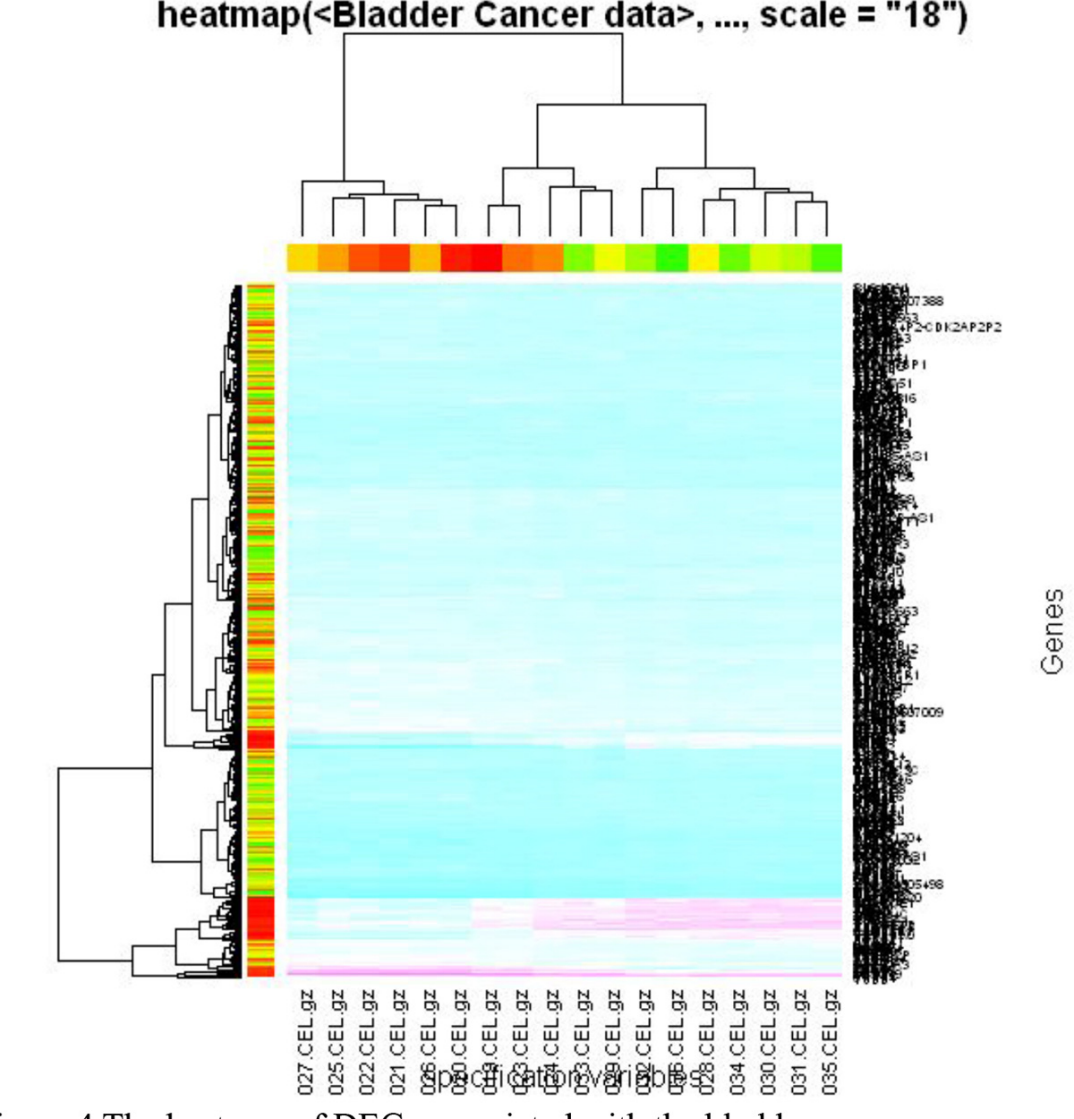
**The heatmap of DEGs associated with the bladder cancer**.

We also investigated the clustering of the DEGs associated with the bladder cancer. The heatmap of the DEGs is showed in Figure 4. From the heatmap, we can see the clustering results of the DEGs. The clustering method can correctly divide the samples into two classes.

### Construction and analysis of two DCNs

**Figure 5:**
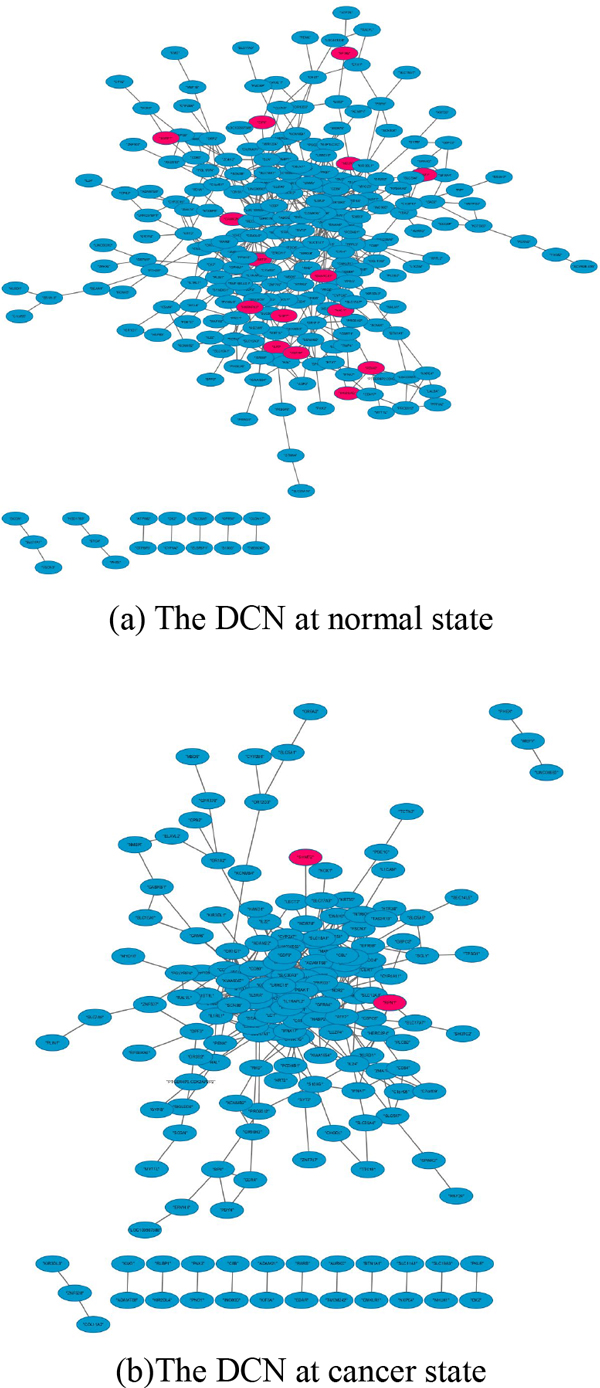
**Two DCNs at normal and cancer state cancer**. The blue ellipses represent down-regulated DEGs and the red ellipses represent up-regulated DEGs. Comparing with the normal DCN, in the cancer DCN, the up-regulated DEGs decrease. In additional, the structure of two differential DCNs change a lot.

**Figure 6:**
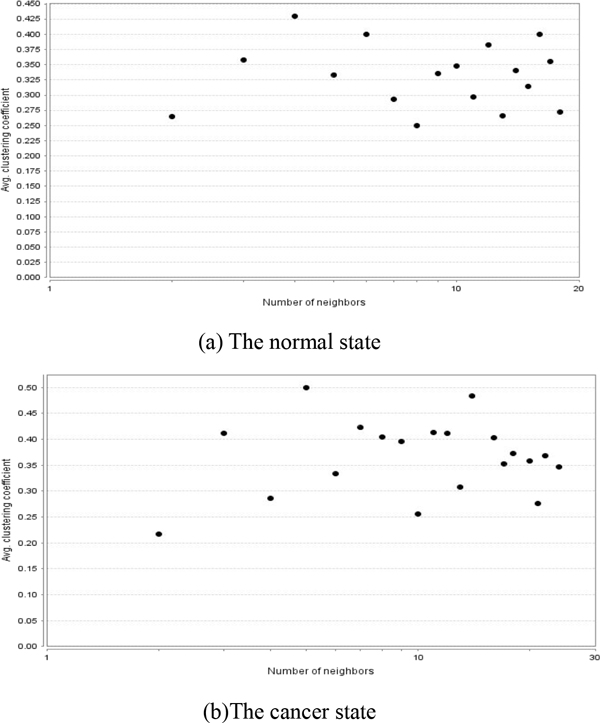
**The avg. clustering coefficient distribution of two different DCNs**. For the normal DCN, the average clustering coefficient distribution is bigger, implying that the cancer DCN has more modules than the normal DCN.

We first calculated a adjacency matrix of DEGs at the normal or cancer state using the method "Pearson correlation" based on the gene expression values. If the adjacency value of a gene pair is greater than 0.8, the two genes will be connected to be an edge of a DCN. We constructed a normal DCN and a cancer DCN (Figure 5) employing the package "WGCNA". We can easily see that in the cancer state, a few up-regulated DEGs take parts in the co-expression relations. Through the comparisons of two different DCNs, we found that the shortest path length distribution (Additional file 3) has a little changes from the normal to the cancer. However, the average clustering coefficient distribution (Figure 6) and the topological coefficients (Additional file 4) under the normal condition differ from those under the cancer conditions, which indicates that the modules in the two different DCNs have a lot of changes.

**Figure 7:**
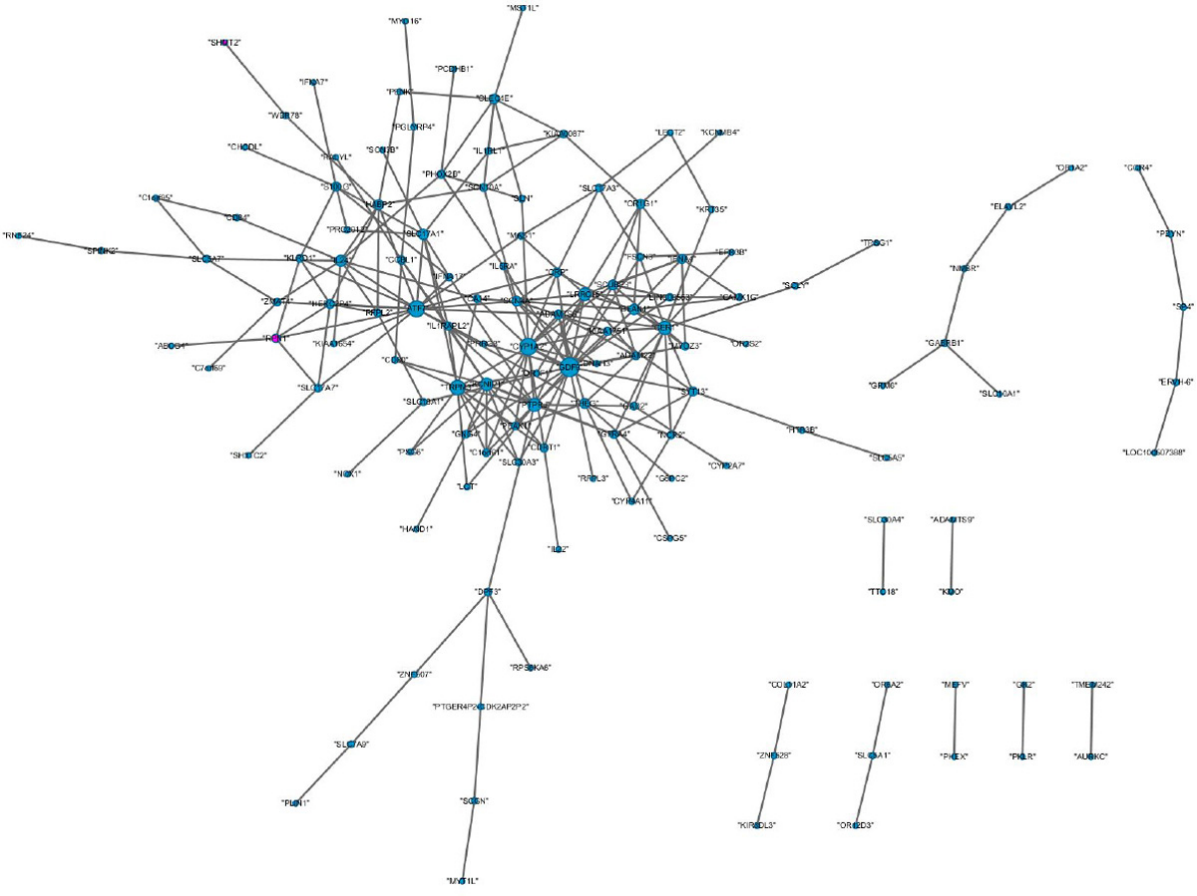
**Differential co-expression subnetwork of two different DCNs**. The blue ellipses represent down-regulated DEGs and the red ellipses represent up-regulated DEGs. The shapes of nodes (genes) grow bigger with the degree of nodes. The 6 biggest nodes represent the hub genes in the subnetwork.

Next, we want to find differential links (edges) between two different DCNs. At first, we set two thresholds T1 and T2. If the correlation (Here is pearson correlation) of a gene pair is less than T1 (Here is set 0.3) at normal state, but is bigger than T2 (Here is set 0.8) at the cancer state, the link of the gene pair is defined as a differential link. We computed the significance of the differential links using permuation test (p-value <2.2e-16). All the selected differential links can compose a differential co-expression subnetwork (Figure 7). In Figure 7, the blue ellipses represent down-regulated DEGs and the red ellipses represent up-regulated DEGs. The shapes of nodes (genes) grow bigger with the degree of nodes. The 6 biggest nodes represent the hub genes: "GDF9", "CYP1A2", "ATF7", "TRPM3", "CER1", "PTPRJ", "KCNIP1", and "LRRC15". These hub genes mainly involve the biological processes: cellular response to stimulus (GO:0051716)(5 genes), regulation of biological process (GO:0050789)(7 genes), response to stimulus (GO:0050896)(6 genes), multi-organism process (GO:0051704)(4 genes), and regulation of localization (GO:0032879)(4 genes). And they mostly take part in five pathways: gamma-Hexachlorocyclohexane degradation (path:hsa00361), Fatty acid metabolism((path:hsa00361)), Adherens junction(path:hsa04520), Tryptophan metabolism(path:hsa00380), and Wnt signaling pathway(path:hsa04310). Among of them, "CYP1A2" and "PTPRJ" have been reported that they are associated with the bladder cancer[21-24].

**Figure 8:**
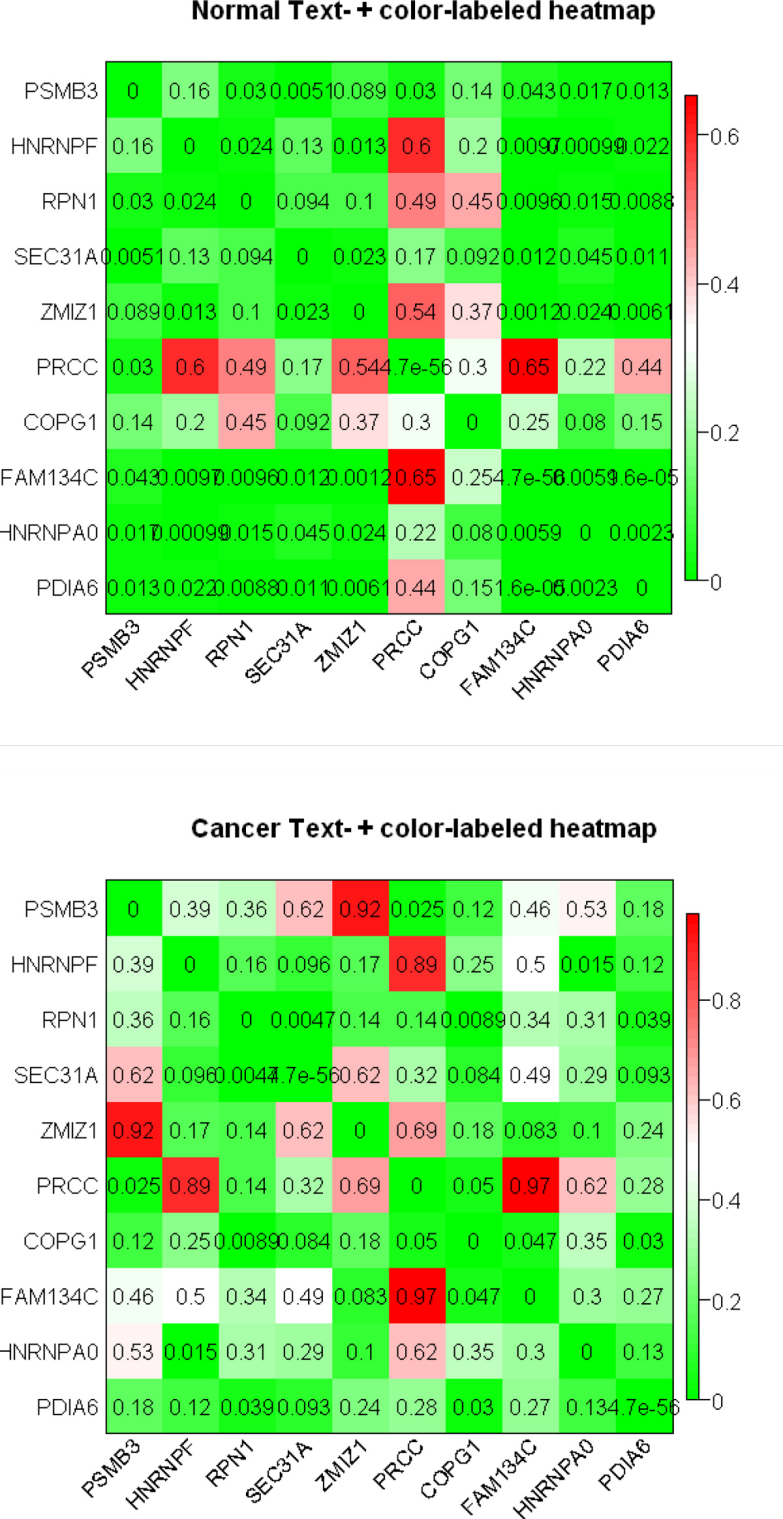
**Part of the modules heatmap of DEGs at two different states**. The colors of grids get deeper with the correlation value and so the genes corresponding to the grids with close colors can be considered to be in the same module.

And then, we dectected the modules of a DCN. There have been some methods using clustering algorithms[25-32]. Here, we adopted another good approach [33]. In order to detect the modules of two DCNs under different conditions, we begun with calculating the topological overlap matrix (TOM)[33] of expression datasets. The topological overlap of two nodes reflects their similarity in terms of the commonality of the nodes they connect to. In order test and verify the inference, we clustered the TOM similarities from two different conditions (Additional file 5). From the Additonal file 5, it is obvious that the modules at two different states change greatly. For observing the module changes, we plotted the module heatmaps of DEGs at two different states and showed parts of the module heatmaps (Figure 8).

## Discussion

The development of molecular markers for tumor classification and expression signatures that predict outcome will greatly improve diagnosis treatment of bladder cancer. We employed the R package "SAMR" to select 392 DEGs including 36 up-regulated and 356 down-regulated. In the GO function enrichment results (Additional file 6 Additional file 7), it is showed that the main functions of the DEGs are cellular physiological precess(24 up-regulated DEGs; 167 down-regulated DEGs) and cellular metabolism (19 up-regulated DEGs; 104 down-regulated DEGs), which is reasonable for the bladder. However, it is not clear why the difference between the number of the up-regulated and the down-regulated is so large.

We used the R packages "WGCNA" to construct two DCNs under different conditions. And we used the tool "Cytoscape" to visualize and analyze the two different DCNs. Some hub genes were found and analyzed in view of bioinformatics. The hub genes of the normal DCN mainly involve the neuroactive ligand-receptor interaction pathway and their GO functions mostly are response to biotic stimulus,response to stimulus and response to external stimulus. The hub genes of the cancer DCN involve the following three KEGG pathways: gamma-Hexachlorocyclohexane degradation, Fatty acid metabolism and Tryptophan metabolism. Their main GO functions are cellular physiological process, surface receptor linked signal transport and signal transduction. In addition, we found some difference of the two DCNs in modules from the clustering plots and the heatmaps. But it is unknown for us that the functions of the different modules, which is our future work.

We found several hub genes from the selected differential co-expression subnetwork of two different DCNs. Two of them have been reported to be associated with the bladder cancer. Then whether are the other hub genes associated with the bladder cancer? It need to be validated through biological experiments.

## Conclusions

In the work, we adopted an integrated strategy to analyzing the bladder cancer-associated genes by combining several R packages, Gene Ontology and KEGG. In the experimental results, it shows that the bladder cancer results from the abnormal signaling pathways caused by many genes. Through the data mining for gene expression microarrays, we found differential co-expression subnetwork and the hub genes of the subnetwork. Through the main GO functions and pathways of the hub genes, we can better understand the development of the bladder cancer, which will support the wet biological experiment and even further promote the prevention, treatment,diagnosis and cure of the bladder cancer in the future.

## Methods

### Selecting differential expressed genes

We adopted the method called "Significance analysis of microarrays (SAM)"[15] to pick out the DEGs. The selection approach is based on analysis of random fluctuations in the data. To account for gene-specific fluctuations, they defined a statistic based on the ratio of change in gene expression to standard deviation in the data for that gene. The "relative difference" *d*(*i*) in gene expression is:

(1) $$d\left(i\right)=\frac{{\bar{x}}_{I}\left(i\right)-{\bar{x}}_{U}\left(i\right)}{s\left(i\right)+s_{0}}$$

Where ${\bar{x}}_{I}\left(i\right)$ and ${\bar{x}}_{U}\left(i\right)$ are defined as the average levels of expression for gene (*i*) in states I and U, respectively. The "gene-specific scatter" *s*(*i*) is the standard deviation of repeated expression measurements:

(2) $$s\left(i\right)=\sqrt{a\left\{\sum_{m}{\left[x_{m}\left(i\right)-{\bar{x}}_{I}\left(i\right)\right]}^{2}+\sum_{n}{\left[x_{n}\left(i\right)-{\bar{x}}_{U}\left(i\right)\right]}^{2}\right\}}$$

Where ∑*_m _*and ∑*_n _*are summations of the expression measurements in states I and U, respectively, *a *= (1/*n*_l _+ 1/*n*_2_)/(*n*_1 _+ *n*_2 _- 2), and *n*_1 _and *n*_2 _are the numbers of measurements in states I and U.

To find significant change in gene expression, genes were ranked by magnitude of their *d*(*i*) values, so that *d*(*i*) was the *i *th largest relative difference. For each of the *N *balanced permutations relative differences *d_p_*(*i*) were also calculated, and the genes were again ranked such that *d_p_*(*i*) was the *i *th largest relative difference for permutation *p*. The expected relative difference, *d_E_*(*i*), was defined as the average over the *N *balanced permutations, $d_{E}\left(i\right)=\sum_{p}d_{p}\left(i\right)/N$.

To identify potentially significant changes in expression, they used a scatter plot of the observed relative difference *d*(*i*) vs. the expected relative difference *d_E_*(*i*). For the vast majority of genes, *d*(*i*) ≅ *d_E_*(*i*), but some genes are represented by points displaced from the *d*(*i*) = *d_E_*(*i*) line by a distance greater than a threshold Δ and these genes were called "significant genes".

The method for setting thresholds provides asymmetric cutoffs for induced and repressed genes. The alternative is the standard t test, which improves a symmetric horizontal cutoff, with *d*(*i*) >*c *for induced genes and *d*(*i*) <*c *for repressed genes.

### Detecting modules in differential co-expression networks

We first needed to construct topological overlap matrices(TOM)[33,34]. The topological overlap is for measuring pair-wise similarity. They start with a network encoded by its corresponding adjacency matrix *A *= [*a_ij_*] which is a symmetric with binary entries. By convention, the diagonal elements are assumed to be zero.

The topological overlap of two nodes reflects their similarity in terms of the commonality of the nodes they connect to. Ravasz et al.[35] define the topological overlap matrix *T *= [*t_ij_*] as follows

(3) tij={lij+aijmin{ki,kj}+1−aijif i≠j1if i=j

Where, $l_{ij}=\sum_{u}a_{iu}a_{uj}$, $k_{i}=\sum_{u}a_{iu}$ and the index *u *runs across all nodes of the network.

Yip and Horvath [33] generalized the TOM of Ravasz et al.[35] by the observation that formula (1) as follows:

(4) $$t_{ij}=\{\begin{array}{ll} \frac{\left|N_{1}(i)\cap N_{1}(j)\right|+a_{ij}}{\min\left\{\left|N_{1}(i)\right|,\left|N_{1}(j)\right|\right\}+1-a_{ij}} & if\;i\neq j\\ 1 & if\;i=j \end{array}$$

Where *N*_1_(*i*) denotes the set of neighbors of *I *excluding *I *itself and |·| denotes the number of elements (cardinality) in its argument. The quantity |*N*_1_(*i*)∩*N*_1_(*j*)| measures the number of common neighbors that nodes *i *and *j *shares whereas |*N*_1_(*i*)| gives the number of neighbors of *i*.

By denoting *N_m_*(*i*)(*with m *> 0) the set of nodes (excluding *i *itself) that are reachable from *i *within a path of length *m*, i.e.,

(5) $$N_{m}\left(i\right):=\left\{j\neq i|\;dist\left(i,j\right)\leq m\right\}$$

Where *dist*(*i*, *j*) is the geodesic distance between *i *and *j*, then a very natural generalization of the TOM can be read as follows

(6) $$t_{ij}^{\left[m\right]}=\{\begin{array}{ll} \frac{\left|N_{m}(i)\cap N_{m}(j)\right|+a_{ij}}{\min\left\{\left|N_{m}(i)\right|,\left|N_{m}(j)\right|\right\}+1-a_{ij}} & if\;i\neq j\\ 1 & if\;i=j \end{array}$$

The matrix $T^{\left[m\right]}=\left[t_{ij}^{\left[m\right]}\right]$ is called the *m *- *th *order generalized topological overlap matrix (GTOMm). This quantity simply measures the agreement between the nodes that are reachable from *i *and from *j *within *m *steps.

## Competing interests

The authors declare that they have no competing interests.

## Authors' contributions

Su-Ping Deng mainly designed all the experiment, did the implementation of the design and wrote all the manuscript. Lin Zhu collected the experimental datasets and preprocessed the datasets. De-Shuang Huang was responsible for the supervision and direction of all the work. Zhu-Hong You revised the manuscript and programmed a part of R resource.

## Supplementary Material

Additional file 1 Click here for file

Additional file 2 Click here for file

Additional file 3 Click here for file

Additional file 4 Click here for file

Additional file 5 Click here for file

Additional file 6 Click here for file

Additional file 7 Click here for file

## Declarations

The publication costs for this article were partly funded by the grants of the National Science Foundation of China(61133010, 61303111, 61373105, 30711140358, 61472280 and 6140050774) and partly supported by the National High-Tec h R&D Program (863) (2014AA021502). And Publication costs for this article were also funded by the corresponding author's institution.

This article has been published as part of *BMC Genomics *Volume 16 Supplement 3, 2015: Selected articles from the 10th International Symposium on Bioinformatics Research and Applications (ISBRA-14): Genomics. The full contents of the supplement are available online at http://www.biomedcentral.com/bmcgenomics/supplements/16/S3.
