# Supplementary figures and images for: Mining the bladder cancer-associated genes by an integrated strategy for the construction and analysis of differential co-expression networks
Source: BMC Genomics. 2015 Jan 29;16(Suppl 3):S4. doi: 10.1186/1471-2164-16-S3-S4 (PMC4331807; doi:10.1186/1471-2164-16-S3-S4)

## Before Normalization

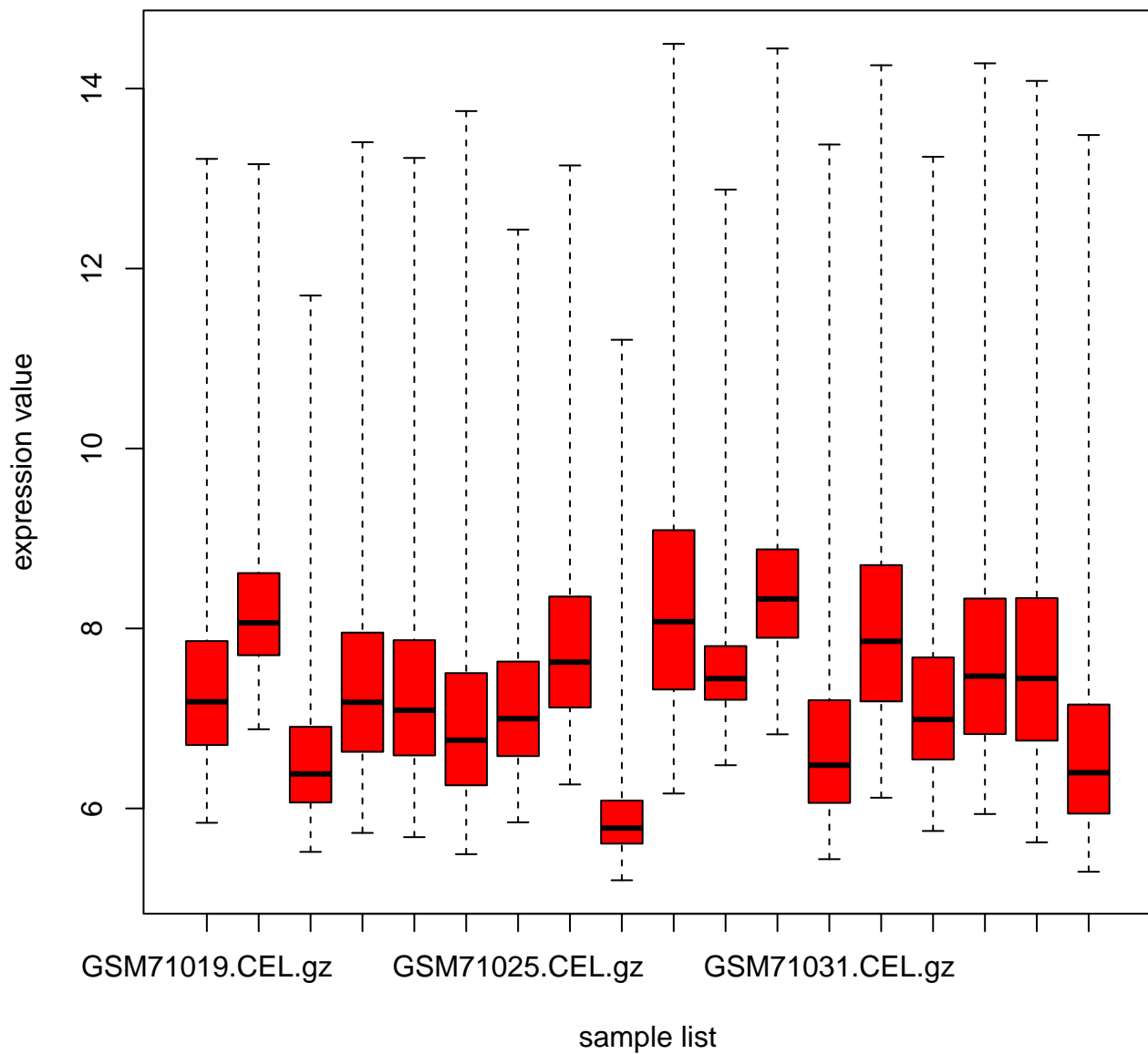

## After Normalization

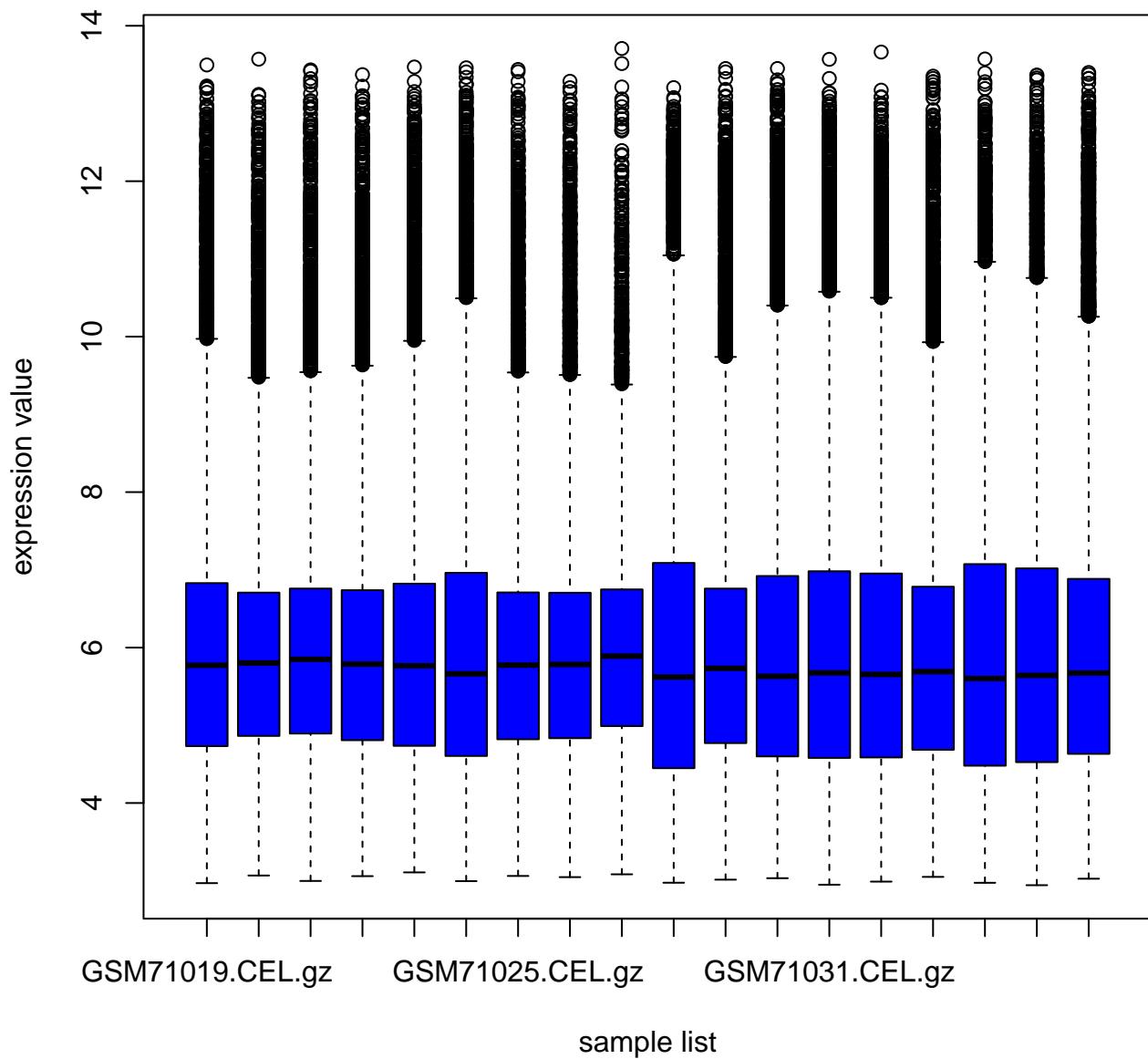

Supplement: Additional file 1 [file 1471-2164-16-S3-S4-S1.pdf]

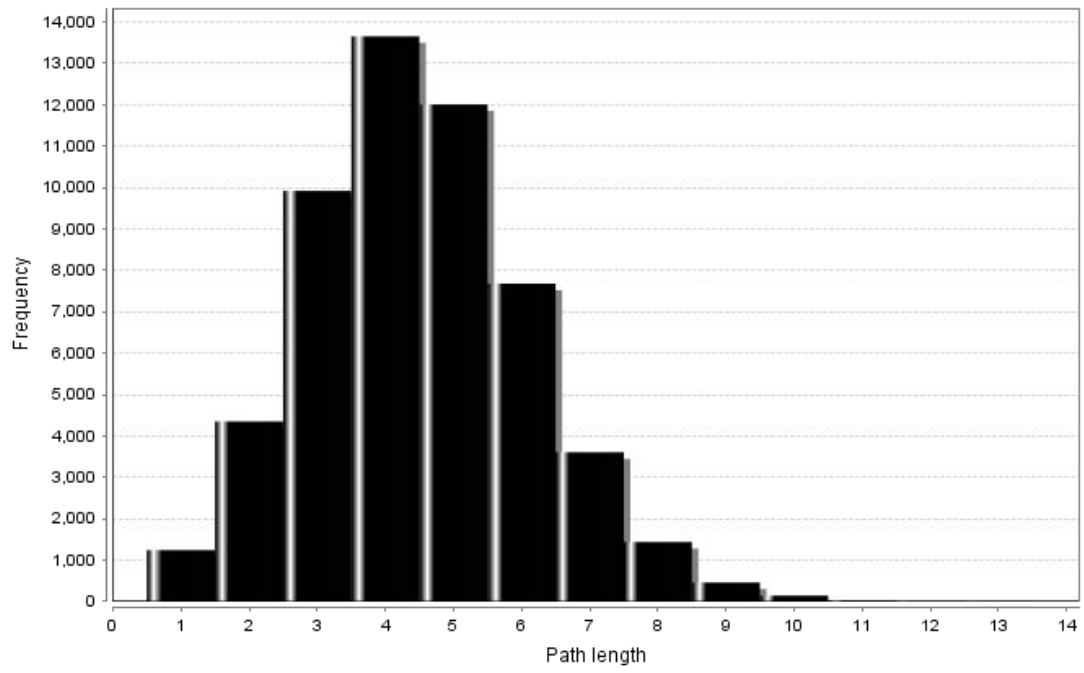

(a) the normal state

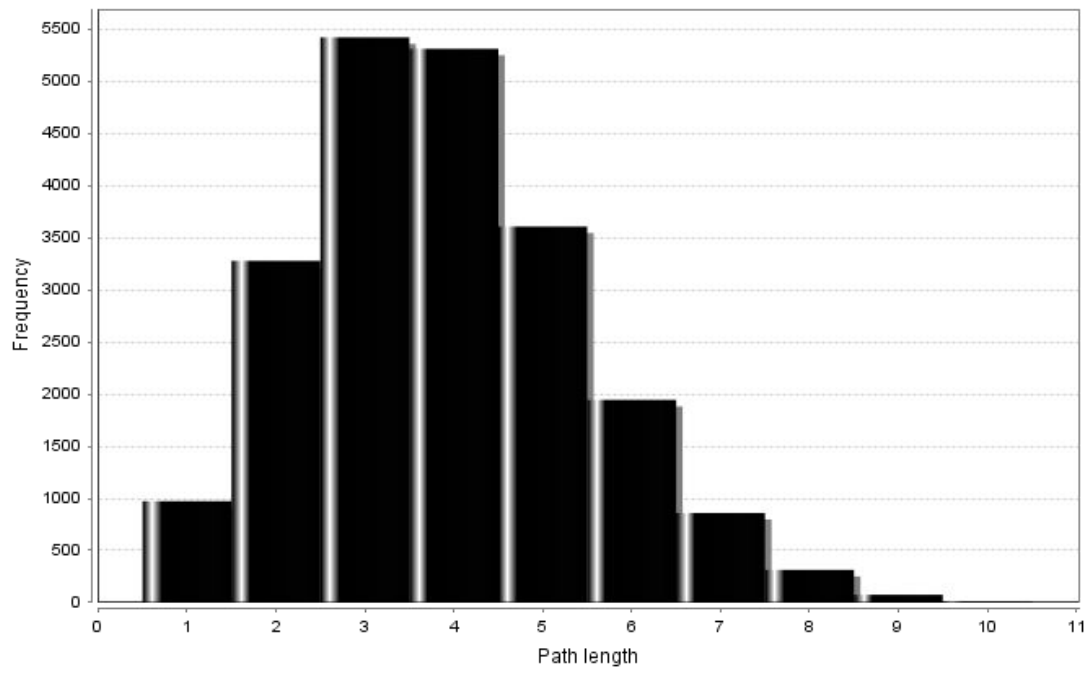

(b) the cancer state

The shortest path distribution of two different DCNs

Supplement: Additional file 3 [file 1471-2164-16-S3-S4-S3.pdf]

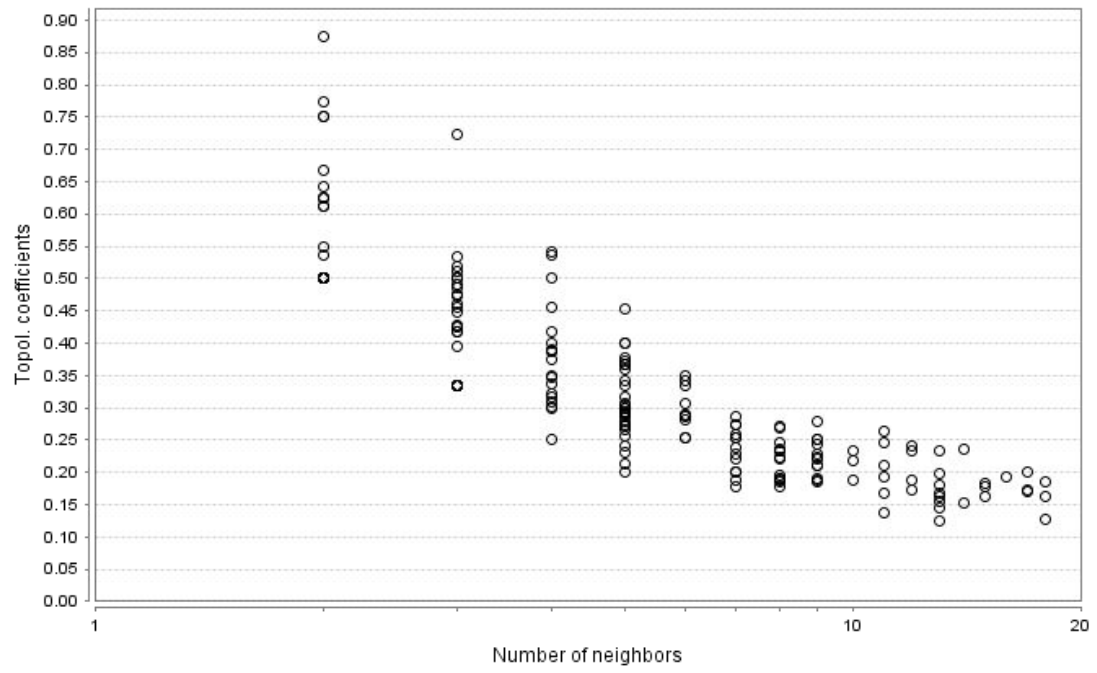

(a) the normal state

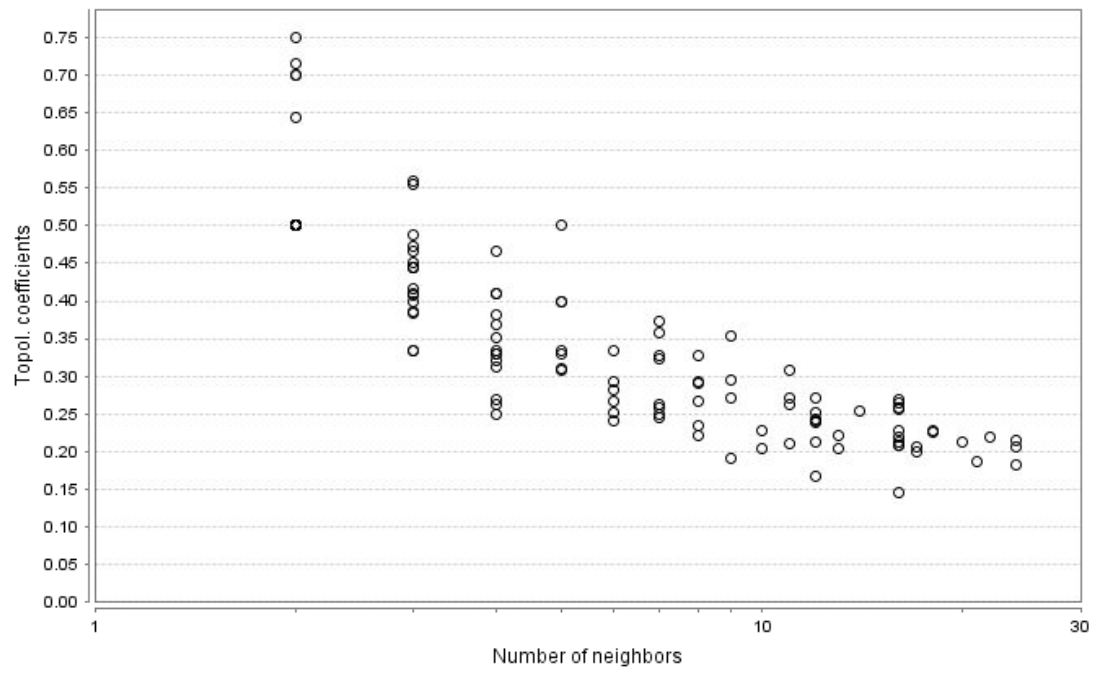

(b) the cancer state

the topological coefficients of two different DCNS

Supplement: Additional file 4 [file 1471-2164-16-S3-S4-S4.pdf]
