# Supplementary material for: Mining the bladder cancer-associated genes by an integrated strategy for the construction and analysis of differential co-expression networks
Source: BMC Genomics. 2015 Jan 29;16(Suppl 3):S4. doi: 10.1186/1471-2164-16-S3-S4 (PMC4331807; doi:10.1186/1471-2164-16-S3-S4)
Supplement: Additional file 5 [file 1471-2164-16-S3-S4-S5.pdf]

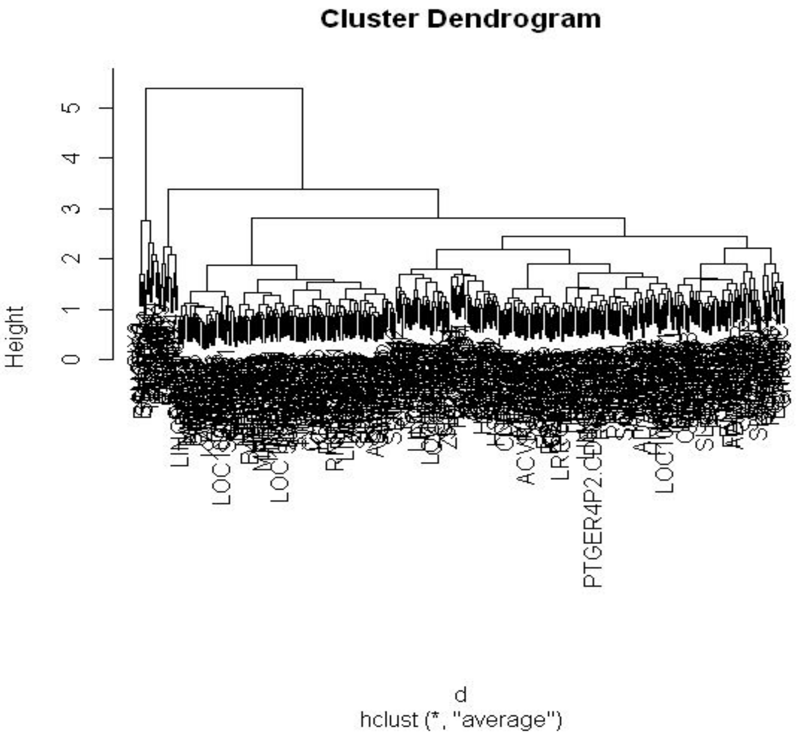


(a) The normal state

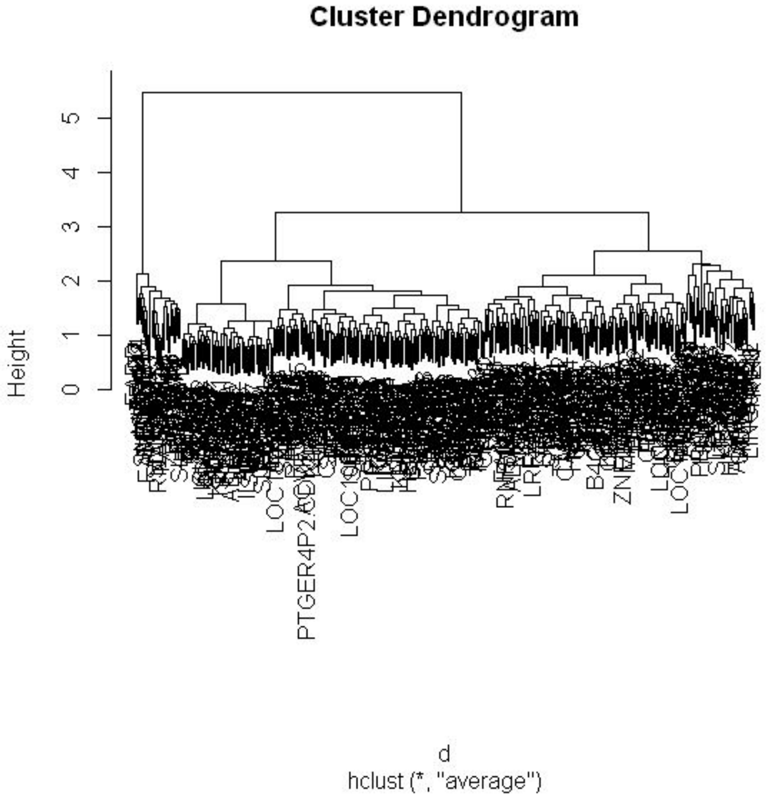


(b)The cancer state

The clustering of the DCGs at two different states.
